# Slow-Oscillation Neurofeedback: A Narrative Review on Clinical Efficacy in Pediatric Settings

**DOI:** 10.3390/bs16030337

**Published:** 2026-02-27

**Authors:** Lea Glaubig, Yasmine Azza, Sabrina Beber, Philipp Silbernagl, Isabel Barradas, Angelika Peer, Reinhard Tschiesner

**Affiliations:** 1 Institute for Biomedicine, Eurac Research, 39100 Bolzano, Italy; lea.glaubig@eurac.edu; 2 Free University of Bozen-Bolzano, Faculty of Education, 39042 Bressanone, Italy; yasmine.azza@unibz.it (Y.A.); sabrina.beber@unibz.it (S.B.); 3 General Hospital Bressanone, Azienda Sanitaria dell’Alto Adige, 39042 Bressanone, Italy; philipp.silbernagl@sabes.it; 4 Free University of Bozen-Bolzano, Faculty of Engineering, 39100 Bolzano, Italy; isabel.barradas@unibz.it (I.B.); angelika.peer@unibz.it (A.P.)

**Keywords:** slow-oscillation neurofeedback, pediatric mental health, self-regulation, neurodevelopmental disorders

## Abstract

Slow-oscillation neurofeedback (NF), encompassing slow cortical potential (SCP), infra-low-frequency (ILF), and infra-slow-fluctuation (ISF) protocols, has gained increasing interest as a non-pharmacological intervention in pediatric mental health and neurodevelopmental care. This narrative review synthesizes peer-reviewed literature on the clinical efficacy of slow-oscillation NF in children and adolescents across various conditions, including attention-deficit/hyperactivity disorder (ADHD), autism spectrum disorder (ASD), epilepsy, tic disorders, and eating-related concerns. SCP NF is the most extensively studied protocol and shows preliminary efficacy in reducing ADHD symptoms, particularly among individuals capable of learning self-regulation. For ASD and other conditions, early evidence from primarily small-scale or uncontrolled studies suggests possible benefits in emotional regulation, impulsivity, and behavioral symptoms, though findings remain mixed and often non-specific. Methodological heterogeneity, including variation in control conditions, training protocols, and outcome measures, limits the comparability of results. ILF and ISF protocols, while promising, are still emerging and require further validation. Overall, slow-oscillation NF appears to offer potential as a personalized therapeutic option for pediatric populations, but robust, well-controlled trials are needed to clarify its clinical utility and optimize its integration into multimodal care.

## 1. Introduction

Neurofeedback (NF) is a type of biofeedback that enables individuals to self-regulate their brain functions by utilizing real-time feedback on brain waves through acoustic, visual, tactile, or combined signals. This technique can be viewed as a form of brain–computer interface (BCI), in which the brain activity is measured via electroencephalography (EEG) and the feedback is provided by a computer interface (e.g., Hinterberger et al., 2005). The NF process often incorporates reward systems to facilitate learning and sustain engagement. For example, successfully modulating brain activity to achieve a predetermined target, as detected by the BCI, may trigger a reward, such as modification of a visual stimulus on the screen. This reinforcement strategy helps individuals learn to regulate their brain activity, enhancing the overall effectiveness of NF training (e.g., Heinrich et al., 2004). By learning to regulate their brain activity, individuals can achieve desired mental states, improve brain function, or reduce pathological symptoms. Neurofeedback is frequently supplemented by the measurement of other physiological parameters (e.g., heart rate, respirate rate, galvanic skin response). This can provide insights into emotional experience and emotion regulation, carrying significant clinical and therapeutic relevance (e.g., Greco et al., 2016; Barradas et al., 2022, 2024, 2025).

As a therapeutic method, NF is particularly applied to conditions associated with dysregulated brainwave activity, such as neurodevelopmental disorders (e.g., attention-deficit/hyperactivity disorder [ADHD], autism spectrum disorder [ASD]). Hence, by targeting these imbalances, NF helps reinforce brainwave patterns, improving self-regulation.

For example, in ADHD, NF training could address characteristic symptoms such as inattention, disorganization, or impulsive hyperactivity by enhancing self-regulation in areas like attention and behavior (e.g., Micoulaud-Franchi et al., 2014). This could include improving the ability to focus and concentrate for extended periods, sustaining effort over time and managing both external and internal impulses (e.g., Pimenta et al., 2021). These improvements could be crucial for functioning in school, social, and family environments (e.g., Moreno-García et al., 2022). On the other hand, in ASD, NF training could help to reduce the severity of core symptoms such as difficulties in social communication, interaction, and motivation (e.g., Konicar et al., 2021; Pineda et al., 2014). It could also alleviate symptoms across various other domains, including attention and learning (e.g., difficulty shifting attention), sensory processing (e.g., auditory hypersensitivity), emotional regulation (e.g., anxiety), physical health (e.g., fatigue/exhaustion), and sleep (e.g., difficulty maintaining sleep), contributing to overall functional improvements (e.g., Rauter et al., 2022). These improvements may be related, in part, to underlying changes in autonomic and sleep regulation, which are increasingly recognized as key aspects of emotional resilience and mental health, highlighting the importance of sleep and autonomic regulation in emotional and psychological functioning (Azza et al., 2020, 2022). More broadly, these effects reflect the intricate interplay of brain regions functioning as an integrated network, an aspect particularly evident when cognitive deficits emerge following localized brain damage (e.g., Beber et al., 2024, 2025).

A variety of approaches are used to implement NF training, each aiming to address distinct brainwave patterns and neurological conditions. These include frequency-based training methods, such as frequency band training (e.g., theta, beta) and slow-oscillation training. While classical frequency band training—such as theta (4–8 Hz) or beta (13–30 Hz)—targets the amplitude of specific oscillations to foster states such as concentration or relaxation (E. Schneider & Krombholz, 2016), slow-oscillation approaches—such as infra-low frequency (ILF) and slow cortical potentials (SCP)—focus on modulating the underlying neural excitability and regulatory stability (E. Schneider & Strauß, 2016; S. Othmer, 2020). In addition, other commonly used approaches include Z-score training (Thatcher et al., 2019), which relies on deviations from normative EEG databases, and sLORETA-based protocols that aim to modulate activity in specific brain regions through source localization (Pascual-Marqui, 2002). In particular, NF paradigms on slow oscillation highlight the crucial role of slow rhythms in the self-regulation of large-scale brain networks by influencing central nervous system organization and regulation (e.g., Larsen & Sherlin, 2013; Grin-Yatsenko et al., 2020). Since slow-oscillation neurofeedback is the focus of this review, the theoretical background and the various forms will now be described in more detail.

### 1.1. Slow Oscillations

Slow oscillations are low-frequency neural activities occurring below 1 Hz, encompassing slow cortical potentials (SCP), infra-low frequency (ILF), and infra-slow fluctuations (ISF). Synchronized post-synaptic potentials in large pyramidal neuron populations are a critical source for SCP (e.g., Elbert, 1993) and play a foundational role in generating ILF and ISF (e.g., Hiltunen et al., 2014). However, ILF and ISF encompass additional mechanisms beyond the post-synaptic activity of pyramidal neurons, involving subcortical structures, glial cells, and vascular responses (e.g., Grin-Yatsenko et al., 2018; Hiltunen et al., 2014; S. Othmer & Othmer, 2024). Their complexity reflects their integrative role in both neural and systemic regulation. Overall, these three oscillation types are fundamental to brain function and physiology, playing a key role in regulating neural excitability, integrating large-scale brain networks, and maintaining balance within the central nervous system (Bazzana et al., 2022; Pineda et al., 2014; Gevensleben et al., 2011).

#### 1.1.1. Slow Cortical Potentials (SCP)

Slow cortical potentials are slow voltage shifts in the EEG, typically below 0.5 Hz, and are closely associated with the excitability of the underlying cortical areas. These shifts in brain activity involve large groups of neurons and are associated with regulatory mechanisms, as well as with behavioral and cognitive processes (Gevensleben et al., 2014; Studer et al., 2014). SCPs are characterized by (e.g., Elbert, 1993; Hinterberger et al., 2003; Studer et al., 2014):

- Negative shifts, associated with depolarization of the neuronal membrane, increasing neuronal excitability and thereby enabling activation of neural pathways (e.g., to perform behavioral or cognitive tasks).
- Positive shifts, linked to hyperpolarization of the neuronal membrane, that reduce cortical excitability and facilitate inhibitory states or rest (e.g., in Go/No-Go paradigm).

In SCP NF, both unipolar and bipolar electrode placements can be used, depending on the specific training protocol and the goal of the session. In unipolar placement, one electrode is placed over a target cortical area (e.g., Cz) and the reference electrode is positioned on a neutral site (e.g., mastoid or earlobe). Hence, it allows the measurement of cortical changes relative to a neutral reference. This configuration is particularly suited for SCP training, as it focuses on detecting slow shifts in cortical excitability (positive or negative) at a specific cortical region. In contrast, bipolar placement, which involves recording the relative potential difference between two active sites, is less suited for protocols targeting localized activity. This configuration introduces additional variability due to interactions between the two electrode sites. Nevertheless, bipolar placement may be used in specific SCP protocols aimed at training the functional interplay between cortical regions.

The Contingent Negative Variation (CNV), also known as the “expectancy wave”, is a type of SCP first described by Walter et al. (1964). It is characterized by a gradual increase in negative electrical potential observed between a warning and an imperative stimulus, during cognitive tasks (e.g., Brunia & van Boxtel, 2001; Prillinger et al., 2022; Walter et al., 1964). This potential is typically observed with a peak in the frontal and central areas (Hamano et al., 1997), reflecting the dynamic interplay of top-down neural mechanisms for response preparation and control (e.g., Prillinger et al., 2022; Stuss & Alexander, 2007).

The negative shift of the CNV signals heightened neural excitability, facilitating readiness for upcoming actions. Furthermore, the CNV exhibits a habituation effect when repetitive stimuli are presented (e.g., Ning et al., 2023), indicating a gradual reduction in anticipatory activity over time. This habituation is linked to the dopaminergic system, as evidenced by studies showing increased CNV amplitude following the administration of methylphenidate—a dopamine-enhancing medication (commonly used to treat ADHD; e.g., Linssen et al., 2011). These findings (e.g., Linssen et al., 2011) suggest the role of dopamine in regulating the anticipatory mechanisms underpinning the CNV. As a type of SCP, the CNV holds particular importance in NF paradigms. SCP-based NF leverages the CNV to train individuals to modulate cortical excitability, thereby improving self-regulation of attention and motor preparation. In conclusion, SCPs have been utilized for decades, with early studies by Elbert et al. (1991) demonstrating their potential for self-regulation training and clinical applications.

#### 1.1.2. Infra-Low Frequency (ILF)

Infra-Low Frequency oscillations, occurring below 0.1 Hz, are ultra-slow dynamics considered to reflect large-scale regulatory activity within brain networks. These interactions among cortical, subcortical and glial-neuronal systems are associated with autonomic regulation, emotional balance, and overall brain state stabilization (S. Othmer & Othmer, 2024; Bazzana et al., 2022; S. Othmer et al., 2011). ILF NF aims to influence these foundational regulatory processes through individually tailored protocols. Electrode placement may be unipolar (e.g., active at T3, reference at mastoid) or bipolar (e.g., T3–T4), depending on whether the goal is to target specific cortical regions or interregional connectivity. Bipolar setups are commonly used to engage cross-hemispheric dynamics.

Unlike traditional NF methods which rely on discrete external reinforcers (e.g., visual or auditory rewards), ILF NF operates on a more dynamic and continuous principle, leveraging the brain’s natural tendency to reconcile discrepancies between expected and perceived signals, thereby enhancing its intrinsic self-regulation mechanisms (e.g., Bazzana et al., 2022; S. Othmer, 2020). This principle fosters a self-regulating loop, in which feedback derived from neural activity helps the brain to gradually adjust toward more stable and functional states.

A central concept in ILF training is the identification of the Optimal Response Frequency (ORF), which refers to the specific frequency within the infra-low range (below 0.1 Hz) at which an individual’s brain demonstrates its greatest capacity for self-regulation. In clinical practice, the concept of ORFs became central to ILF NF from 1996 onwards, as it was observed that training at individually tailored frequencies could enhance therapeutic effects (S. Othmer, 2020). The ORF is unique to each person and is identified by observing their responses at various frequencies.

The ORF is thought to reflect the brain’s most efficient operating point for engaging neural networks and stabilizing arousal and emotional states (e.g., S. F. Othmer, 2017; Rauter et al., 2022). ILF NF has been associated with therapeutic effects in conditions such as ADHD (Prinz, 2015), ASD (Esmaeilzadeh Kanafgourabi et al., 2023; Rauter et al., 2022), and emotional dysregulation (Grin-Yatsenko et al., 2020; Tschiesner, 2023). Although evidence remains largely observational, the clinical application of ILF training has expanded steadily since the late 1990s, supported by reports of improved behavioral regulation and physiological stability (Grin-Yatsenko et al., 2020; Solberg & Solberg, 2022).

#### 1.1.3. Infra-Slow Fluctuations (ISF)

Infra-Slow Fluctuations represent slow neural oscillations, occurring within the 0.01–0.1 Hz range (Aladjalova, 1957). These rhythms are believed to underlie slow regulatory mechanisms in the brain, linking neuronal, glial, and vascular systems and contributing to the modulation of brain state transitions, resting-state connectivity, and large-scale functional organization (Aladjalova, 1957; Hiltunen et al., 2014; S. Othmer & Othmer, 2024).

ISFs are thought to play a critical role in maintaining global brain dynamics by supporting long-term processes like functional connectivity and overall brain state transitions (e.g., Hiltunen et al., 2014). These fluctuations may regulate resting-state networks of the brain, acting as a foundational rhythmic process that helps organize brain function, ensuring it remains fluid and timely (Watson, 2018; Infraslow Associates, 2024). Additionally, brain imaging studies demonstrated that resting-state blood-oxygen-level-dependent (BOLD) signals are divided into specific networks, with frequencies ranging from 0.007 to 0.438 Hz, further connecting ISFs to the brain’s core functional architecture (Kajimura et al., 2023).

ISFs contribute to the brain’s physiological and functional regulation (e.g., Raut et al., 2021) and support global connectivity by synchronizing activity across distant brain regions. This synchronization enables cohesive and coordinated brain function, which underpins complex behaviors and regulatory mechanisms (Hiltunen et al., 2014; Raut et al., 2021; Smith, 2013; Kropotov, 2022). Preliminary findings suggest that ISF NF may help stabilize ultra-slow neural activity and promote self-regulatory capacities, particularly in individuals with neurodevelopmental and psychological disorders (Smith, 2013). By modulating these slow fluctuations, ISF NF may facilitate smoother transitions between brain states and support improved functional integration. While still an emerging technique, ISF NF appears promising for restoring balance in individuals experiencing dysregulated neural dynamics. In ISF neurofeedback, bipolar electrode montages and full-band EEG setups, typically using the 10–20 system, are employed to monitor and train these ultra-slow dynamics.

#### 1.1.4. Summary and Differences Between Protocols

While the SCP, ILF, and ISF neurofeedback protocols all operate in the slow-frequency range (<1 Hz), they differ in their specific frequency targets, electrode configurations, and proposed mechanisms of action (see Table 1). SCP training focuses on cortical excitability through event-related shifts below 0.5 Hz, typically using unipolar placements to modulate task-related potentials such as the Contingent Negative Variation (e.g., Gevensleben et al., 2014; Studer et al., 2014). ILF neurofeedback targets even slower oscillations (<0.1 Hz) and emphasizes emotional and autonomic regulation via individually optimized frequencies, often applied with bipolar montages (e.g., S. Othmer & Othmer, 2024; Bazzana et al., 2022). ISF neurofeedback operates in the 0.01–0.1 Hz range, using full-band EEG to support large-scale brain dynamics and long-range functional integration (e.g., Hiltunen et al., 2014; Aladjalova, 1957).

These protocols reflect distinct conceptualizations of self-regulation in the brain—from phasic, task-related control (SCP) to continuous stabilization of arousal and connectivity (ILF, ISF). While clinical observations are encouraging, further empirical studies are needed to clarify the differential mechanisms and therapeutic outcomes associated with each approach.

### 1.2. Aim of the Review

This study focuses on children and adolescents as the target population, addressing the critical need for effective and individualized therapeutic options in pediatric neurodevelopmental and mental health contexts. Neurofeedback as a non-invasive, non-pharmacological approach holds promise as a targeted intervention to support self-regulation and improve functioning in this population. Conventional treatments, including pharmacological interventions, can often be insufficient. Many children do not respond to prescribed therapies, or they experience adverse effects (Coleman et al., 2019; Davico et al., 2023; Schweren et al., 2019; Spencer et al., 2002; Wigal, 2009). Given the limited efficacy and potential adverse effects of conventional treatments in pediatric populations, NF stands out as a promising alternative. Its ability to offer personalized interventions provides an opportunity to address symptoms associated with conditions such as neurodevelopmental disorders.

Despite the availability of various NF training, individual differences in developmental trajectories, as well as the complexity of pathological conditions in pediatric settings, pose challenges to achieving consistent outcomes. Moreover, information and protocols related to slow oscillations in neurofeedback remain poorly understood, particularly regarding their application and impact in pediatric settings. This narrative review aims to investigate the potential and limitations of slow-oscillation NF approaches to contribute to the expansion of the therapeutic landscape for children and adolescents with neurodevelopmental and mental health disorders. The current manuscript is designed specifically as a narrative review of clinical outcome studies. A detailed analysis of etiogenesis falls outside the intended scope of this paper, which prioritizes treatment efficacy over process research.

## 2. Method

A structured search of peer-reviewed publications was conducted in the electronic databases PubMed and Google Scholar through May 2025 to identify relevant literature. Pre-defined keywords, “Neurofeedback”, “Slow”, “Low”, “Infra-Low-Frequency”, and “Infra-slow”, were consistently paired with “Children” in the literature search. Duplicates were removed, and abstracts were screened to exclude studies with misleading terminology (e.g., “Low” referring to low-functioning autism).

**Figure 1:**
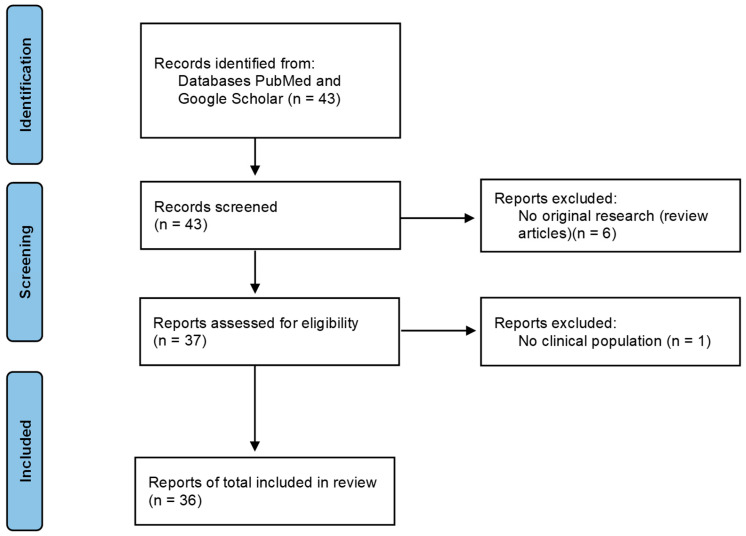
PRISMA process of literature selection.

As shown in Figure 1, this process yielded 43 potentially relevant articles, including six review papers, which were excluded from further analysis. One additional study involving only healthy participants was also excluded, as it did not match the clinical focus of this review. The final dataset included 36 original research articles or case reports, which were analyzed in terms of clinical population, participant age and sex, study design, neurofeedback modality, and clinical change (see Section 3 for an overview of reviewed study details). Results were organized by diagnostic category and presented in chronological order.

## 3. Results

**Figure 2:**
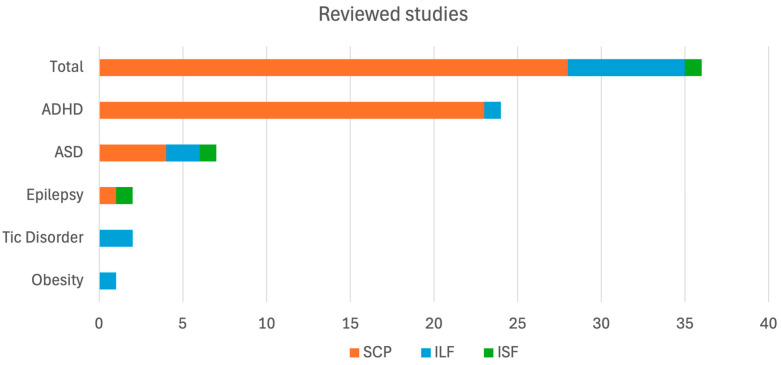
Reviewed studies categorized by target conditions and neurofeedback protocols. Notes: This frequency plot illustrates the total count of reviewed studies and the relative distribution of studies targeting specific conditions, including obesity, tic disorder, epilepsy, autism spectrum disorder (ASD), and attention deficit hyperactivity disorder (ADHD), categorized by the utilized neurofeedback protocol. Protocols are represented by color: orange indicates slow cortical potential (SCP) neurofeedback, blue represents infra-low frequency (ILF) neurofeedback, and green signifies infra-slow-fluctuation (ISF) neurofeedback.

The distribution of reviewed studies across the total dataset, categorized by target conditions and neurofeedback protocols, is illustrated in Figure 2, while Table 2 shows the details of the individual studies.

### 3.1. Studies on Attention-Deficit/Hyperactivity Disorder

#### 3.1.1. Efficacy of SCP Neurofeedback and Comparison with Theta/Beta NF Training

Research articles on ADHD were the most prevalent in this review, with 24 studies specifically investigating the application of slow NF in children. Apart from one ILF study (H. Schneider et al., 2022), all of these studies utilized SCP protocols.

Early research by Strehl et al. (2006) demonstrated in a single-arm study that children with ADHD could learn to regulate their SCPs. A comparison of neurophysiological data before and after treatment showed that the children acquired the ability to regulate negative SCPs. Behavioural outcomes included improvements in cognitive and attentional domains, as well as reductions in behavioural symptoms based on parent and teacher ratings. Importantly improvements were predicted by training performance, i.e., how well the child learned to control their SCPs. All changes remained stable at the 6-month follow-up. Albrecht et al. (2017) also demonstrated the long-term efficacy of SCP neurofeedback in a pre-/post 6-month follow-up assessment, albeit also without a control group design.

In the initial comparative studies on this research topic, the efficacy of SCP NF was evaluated alongside theta/beta NF training (Leins et al., 2006, 2007). Both NF protocols led to clinical improvements, particularly in intelligence and attention measures. Parent and teacher ratings also indicated reduced behavioral problems. However, since no significant differences were found between SCP and theta/beta protocols, the observed improvements may reflect general NF effect rather than protocol-specific mechanisms.

#### 3.1.2. Comparison of SCP Neurofeedback with Other Forms of Therapy

A research team (Drechsler et al., 2007; Doehnert et al., 2008) compared SCP-NF with group therapy aimed at improving self-regulation in children with ADHD. While teacher and parent ratings showed improvements in attention and cognition only in the SCP group, EEG analyses revealed no significant differences in SCP self-regulation between groups during feedback positivity and negativity trials. Consequently, the neurophysiological mechanisms that lead to the reduction of ADHD symptoms remained unclear. This contrasts with findings from Strehl et al. (2006), where SCP regulation was more clearly demonstrated. Drechsler et al. (2007) observed substantial individual differences in the ability to regulate brain activity, as assessed during no-feedback transfer trials. Based on their performance in these trials, participants were classified as “good performers” (those who could distinguish between positivity and negativity trials) and “poor performers”. Crucially, only the good performer exhibited significant reductions in hyperactivity, suggesting that successful SCP regulation during the transfer trials may be essential for achieving notable benefits.

In a large, randomized control trial (RCT; *n* = 102), Gevensleben et al. (2009b) compared a combined SCP and theta/beta NF protocol with an active control group (computer-based attention training) in children with ADHD, thereby demonstrating the efficacy of NF. Gevensleben et al. (2009a) were also able to show that NF leads to measurable neural changes in children with ADHD, which are associated with an improvement in symptoms, whereas this was not the case for computer-based attention training. Follow-up analyses from the same trial showed that the observed improvements, particularly in attention and executive functioning, were stable at 6 months (Gevensleben et al., 2010; Wangler et al., 2011). Parent ratings in a later study also favored SCP over theta/beta training. However, Heinrich et al. (2020) reported that the general effects could not be replicated in a new cohort, raising questions about the robustness of previous findings. While the study was able to show significant improvements in ADHD symptoms compared to the pre-training baseline, it failed to demonstrate the specific superiority of neurofeedback over the control group (a computer-based attention training). The specificity of the effects of SCP neurofeedback has been called into question by further recent research. A large RCT with 202 participants found no significant difference between SCP NF and working memory training, although both performed better at post-test than treatment-as-usual (Hasslinger et al., 2022a, 2022b). These results imply that while SCP may contribute to therapeutic improvement, it may not be uniquely effective when compared to other structured interventions. Christiansen et al. (2014) also demonstrated that Slow Cortical Potential (SCP) neurofeedback is not superior to self-management (behavioral therapy) in a naturalistic setting involving patients who were also receiving medication, albeit in a high-frequency outpatient context. Similarly, Minder et al. (2018) provided evidence for the comparable effectiveness of SCP and computerized cognitive training (targeting attention, working memory, or inhibition). Aggensteiner et al. (2019) compared the outcomes of SCP neurofeedback with EMG biofeedback and found large effect sizes for both groups at the 6-month follow-up; however, they were likewise unable to identify any significant differences between the groups.

In contrast, Strehl et al. (2017) reached a different conclusion in a multicenter RCT that compared SCP NF with electromyography (EMG) biofeedback. Children in the SCP group showed significantly greater reductions in ADHD symptoms, supporting SCP-specific efficacy. Similarly, Baumeister et al. (2018, 2019) used EMG-Feedback as a control and found greater clinical improvement in the SCP group of young adolescents with ADHD. Overall, SCP NF was shown to provide some benefits compared to group therapy and EMG feedback, while individual differences may play a crucial role in determining significant improvements.

#### 3.1.3. Predictors of Neurofeedback Learning

One study specifically investigated the predictive factors for successful NF training (Okumura et al., 2019). Participants were divided into “learners”, defined as those who increased the number of trials with positive SCP shifts (indicating they successfully learned to regulate their SCPs), and “non-learners”. Notably, learners demonstrated higher clinical improvement rates, although changes on the ADHD rating scale were minimal across the sample, and overall symptom reduction was not significant. The study also found that successful SCP regulation was more likely in children with stronger executive functions and older age, suggesting that maturation-related factors may influence NF outcomes. This is consistent with prior research suggesting that executive functions are often delayed in children with ADHD (Berger et al., 2013).

Neurophysiological markers were also investigated as predictors. EEG biomarkers, such as contingent negative variation (CNV) and cue-related P3 activity, have been studied in relation to ADHD symptom severity and NF outcomes. These markers were shown to be partially predictive of treatment response to SCP NF, with increases in neural preparation associated with clinical improvement (Aggensteiner et al., 2021). Although the overall neurophysiological effects of SCP NF were modest, these findings suggest that EEG-based indicators may support individualized treatment planning in NF interventions.

Furthermore, the study by Zuberer et al. (2018) showed that older children with ADHD in particular benefit more from SCP NF than younger ones. Constant Methylphenidate medication also positively influenced the effectiveness of NF, while higher IQ was similarly associated with better outcomes.

### 3.2. Studies on Autism Spectrum Disorder

Out of the 36 studies identified in this review, seven investigated the efficacy of slow-oscillation NF in individuals with ASD (see Table 2).

#### 3.2.1. SCP Neurofeedback in Autism Spectrum Disorder

More or less the same research group (Konicar et al., 2021; Prillinger et al., 2022; Werneck-Rohrer et al., 2022; Klöbl et al., 2023) extensively explored the application of SCP NF in adolescents with ASD. Evidence suggests that improvements in executive functioning are often tied to individual differences in impulsivity control. Specifically, Prillinger et al. (2022) reported that participants with greater deficits in impulsivity control showed the greatest improvements following the SCP training.

Clinical comparisons between SCP NF training and standard treatments (Klöbl et al., 2023) indicate that while behavioral improvements occur in both settings, they remain largely unspecific and focused on affective domains. These included changes related to emotional regulation and reactivity, which, while not directly targeting core ASD symptoms, are functionally related to social interaction. The observed improvements were accompanied by altered activation patterns in brain regions commonly implicated in ASD, including the anterior cingulate cortex and the right temporal gyrus (Klöbl et al., 2023).

The study by Werneck-Rohrer et al. (2022) examined gender differences in response to NF among adolescents with ASD. Both male and female adolescents showed slight improvements in emotion recognition and cognitive flexibility after NF compared to before the intervention. Although behavioral gains, such as faster reaction times and fewer errors, were greater in male participants, these differences were not statistically significant. Specifically, females showed smaller improvements in externalizing and overall symptoms, but no improvement in internalizing problems. In contrast, males exhibited higher baseline levels of problems in all areas, which may have contributed to the observed improvements. Interestingly, caregiver ratings indicated greater improvements in overall functioning for females, while for males these ratings showed improvements in internalizing and total problem behavior.

Among the seven studies reviewed, only the work by Konicar et al. (2021) reported robust treatment-specific effects of SCP NF on ASD symptoms. Using Bayesian analysis, the authors found significant improvements in ASD-related parameters in the NF group compared to an active control group, that received counseling (Konicar et al., 2021).

#### 3.2.2. ILF Neurofeedback in Autism Spectrum Disorder

In addition to SCP-based protocols, ILF has been applied in the treatment of ASD in youth. Esmaeilzadeh Kanafgourabi et al. (2023) reported that ILF NF led to improvements in executive functions, particularly inhibitory control, in high-functioning adolescents with ASD. Neurophysiological changes were observed in the form of increased absolute alpha and beta power at follow-up. The brainwave measures are associated with enhanced cognitive functioning. These findings suggest that ILF NF may be effective in addressing executive dysfunction, a hallmark of ASD. Further support for ILF NF in ASD comes from a case report by Rauter et al. (2022), which described the treatment of a 5-year-old boy with ASD. The intervention led to significant reductions in symptom severity across various categories, with the strongest effects seen in physical symptoms (80% reduction) and sleep disturbances (77% reduction). Behavioral symptoms, such as stimming and self-injurious behavior, showed more modest improvements (15% reduction). Overall, the case highlighted meaningful improvements in both the child’s and the family’s quality of life, reinforcing the potential of ILF NF to address a broad range of ASD-related changes, particularly somatic and sleep-related symptoms.

### 3.3. Studies on Other Disorders

#### 3.3.1. Epilepsy

Research on slow-oscillation NF in epilepsy remains limited but includes both controlled and case studies. In an RCT, Morales-Quezada et al. (2019) compared sensorimotor rhythm (SMR) NF, SCP NF, and sham treatment in a three-arm design involving 44 adolescents diagnosed with focal epilepsy. Results showed improved attention following SMR NF, and both NF protocols were associated with reduced seizure frequency. However, self-reported improvements in quality of life were attributed in part to placebo effects, and seizure-related outcomes were not maintained at follow-up. The results suggest a potential role for SMR NF, while SCP NF showed limited sustained benefits in this context.

Additional case-based evidence comes from Legarda et al. (2011), who reported on three pediatric patients with epilepsy undergoing ISF NF. Across the cases, participants showed improvements in headaches and sleep, medication reduction, and seizure control, including one adolescent who became seizure-free after training. While the results are encouraging, the case-based nature of the evidence limits the extent to which the findings can be generalized.

#### 3.3.2. Studies on Tic Disorders

Two studies have explored the application of slow-oscillation NF in pediatric tic disorders. In a case report, Prinz (2015) described two adolescents with tic disorders who received ILF-NF training. Both participants experienced reductions in motor tics and impulsivity, and one showed improved ability to concentrate. The author concluded that ILF NF may support self-regulatory processes underlying tic symptomatology.

In a broader summary of clinical cases, Solberg and Solberg (2022) shared outcomes from 100 children and adolescents treated with ILF NF for tic disorders, especially also for Tourette’s. The authors noted reductions in severe symptoms and improved overall functioning following the treatment. The authors concluded that most children benefited from NF training, recommending it as a complementary treatment for tic disorders (Solberg & Solberg, 2022).

#### 3.3.3. Studies on Eating Disorders

Slow-oscillation NF has also been explored for addressing eating disorders. In a pilot study (Chirita-Emandi & Puiu, 2014), twelve children underwent 20 ILF NF sessions. Both the intervention group and the control group received traditional lifestyle advice to manage their weight. Contrary to expectations, children in the NF group lost less weight than those in the control group. Nevertheless, subjective improvements were reported, including reduced snacking, improved satiety, better attention capacity, and enhanced sleep.

## 4. Discussion

The present review aimed to summarize and synthesize findings on the clinical efficacy of slow-oscillation NF—including SCP, ILF, and ISF protocols—in pediatric populations. Specifically, we sought to address whether these NF approaches effectively reduce symptoms across various childhood and adolescent disorders, including ADHD, ASD, epilepsy, tic disorders, and eating disorders. To that end, we collected peer-reviewed studies addressing this question and summarized the findings across methodologies and clinical patterns. Across the included studies, SCP NF emerged as the most extensively studied and supported protocol, particularly in the treatment of ADHD. In contrast, evidence for ILF and ISF remains limited, with findings primarily derived from small-scale trials and case reports. While some studies suggest potential benefits across conditions such as ASD, epilepsy, and tic disorders, future research is needed to establish their efficacy and optimize implementation.

### 4.1. Clinical Insights into Slow-Oscillation Neurofeedback Across Pediatric Disorders

#### 4.1.1. Attention-Deficit/Hyperactivity Disorder

Among the disorders reviewed, ADHD has been the most extensively studied condition. The majority of studies we reviewed (see Figure 2) focused on SCP NF, which aims to train self-regulation of cortical excitability, often disrupted in individuals with ADHD. SCPs reflect shifts in neural excitability and have been associated with deficits in attentional control of individuals with ADHD (Rockstroh et al., 1990). In SCP training, participants learn to produce negative or positive shifts in SCPs, which are believed to correspond to increased cortical activation (e.g., during preparation for cognitive tasks) or cortical inhibition, respectively (Studer et al., 2014). By enhancing the ability to modulate these states, SNP training may help to normalize the atypical arousal regulation frequently observed in ADHD.

Eighteen RCTs (see Table 2) have been applied to investigate the feasibility of SCP-NF for ADHD, showing its potential to reduce symptom severity. However, the findings also suggest that the effectiveness of SCP training is not universal. Its success appears to be closely tied to the individual’s ability to learn and apply SCP regulation strategies. Studies consistently show that participants classified as “learners” tend to exhibit greater symptom improvement. These findings emphasize the importance of personalized interventions and consideration of individual differences, such as executive functioning, motivation, and age. While less research exists on ILF (H. Schneider et al., 2022) and ISF protocols for ADHD, preliminary findings suggest these approaches may hold promise, although methodological variability currently limits comparability and interpretation.

#### 4.1.2. Autism Spectrum Disorder

Even though research on slow-oscillation NF for ASD is still in its infancy, with studies emerging only in recent years, ILF and SCP protocols have shown initial potential in addressing aspects of ASD symptomatology, particularly emotional regulation, impulsivity, and cognitive flexibility. As previously noted (Section 3.2.1), Klöbl et al. (2023) found affective improvements and corresponding changes in brain activation following SCP NF in adolescents with ASD. Although these effects were not specific to the NF group, task-related activation correlated with emotional improvements, suggesting a potential neurophysiological mechanism underlying these changes. However, these changes were also observed in the treatment-as-usual group, and no statistically significant differences between groups were found. The author did note that task-related brain activation in these regions correlated with improvements in emotional functioning, hinting at a potential neurophysiological mechanism underlying these effects, but not necessarily a protocol-specific effect of NF.

Other studies (e.g., Prillinger et al., 2022; Werneck-Rohrer et al., 2022) suggest that individual factors such as baseline impulsivity or executive functioning may moderate treatment response to slow-oscillation NF in individuals with ASD. Notably, ILF NF has been associated with improvements in inhibitory control and sensory regulation, but again, the data stem from small samples, lack robust control comparisons, and need replication in larger sample sets.

Only one study systematically examined gender differences in NF response (Werneck-Rohrer et al., 2022), suggesting slightly more pronounced improvements in emotional and behavioral functioning for males. However, females were rated as showing greater improvements in overall functioning by caregivers. Despite these insights, most slow-frequency NF studies to date do not report gender-specific analyses, limiting conclusions regarding differential effects. We also observed that several of the reports we identified—particularly those regarding ASD—were based on studies conducted exclusively on young males. Given the known gender differences in symptom profiles and neurodevelopmental trajectories in psychiatric disorders affecting children and adolescents, future studies should explicitly investigate how gender may influence neurofeedback efficacy and tailor protocols accordingly.

Taken together, these findings align with the literature showing that NF may support regulatory capacities in ASD (Pineda et al., 2014; Coben et al., 2010), but the specificity of effects, particularly in slow-oscillation protocols remain unclear.

The observed improvements in affective functioning align with the neurodevelopmental models emphasizing emotion dysregulation as a core transdiagnostic feature of ASD (Mazefsky et al., 2013). The correlation between NF training and emotional improvements (e.g., Klöbl et al., 2023) provides a promising base for exploring how NF may influence emotional regulation systems that are altered in ASD. The evidence to date however makes it difficult to attribute improvements to solely the NF protocol. Future research should include better-powered RCT’s, clearly defined outcome measures, and comparisons between different NF protocols.

#### 4.1.3. Other Disorders

Beyond ADHD and ASD, slow-oscillation NF has been studied in a small number of other disorders.

In epilepsy, both SCP and SMR NF have shown potential in reducing seizure frequency and improving attention (Morales-Quezada et al., 2019). However, the variability of follow-up data suggests that the findings should be interpreted with caution. Case studies on ISF NF (Legarda et al., 2011) suggest improvements in sleep, seizure activity, and medication use.

In tic disorders, further case reports suggest that ILF NF may reduce symptom severity and enhance self-regulatory functioning (Prinz, 2015; Solberg & Solberg, 2022). Evidence in the context of eating disorders is even more scarce. While neurofeedback has been studied more extensively in adults with eating disorders (e.g., Winkeler et al., 2022), evidence for its use in children remains limited. Within this review, only one pilot study (Chirita-Emandi & Puiu, 2014) addressed excessive eating and snacking in the context of pediatric obesity. They reported subjective improvements in appetite regulation, attention, and sleep, though the intervention group showed less weight loss than controls.

In sum, early findings suggest possible regulatory effects of NF in various pediatric disorders, but the study design limits the interpretability of results.

### 4.2. Methodological Limitations

Several methodological challenges limit the interpretation of findings in this review. First, the diversity of control conditions in NF studies—ranging from sham feedback to alternative treatments such as cognitive tasks or no intervention—complicates direct comparisons and may introduce non-specific effects. Second, inconsistencies in NF protocols across studies, including session frequency, feedback modalities, and outcome measures, further limit comparability of results.

Among the three reviewed protocols, SCP NF is the only one with relatively consistent methodology and sufficient data to support preliminary conclusions about its clinical utility. In contrast, ILF and ISF NF remain underexplored in peer-reviewed research and are often reported in the form of uncontrolled case reports.

Most of the studies reviewed focus on attention-related disorders such as ADHD and ASD, which may be partially due to the long-standing use of neurofeedback for attentional control. In contrast, emotional and motivational dysregulation as seen in mood, eating, or tic disorders has been less frequently targeted, possibly due to the greater complexity of training higher-order cognitive and affective processes through feedback-based learning.

Furthermore, it became clear that the revised literature focuses primarily on the treatment of clinical symptoms, while the neurodevelopmental underpinnings and the effects of slow-oscillation NF treatment on the ongoing development of the nervous system remain underexplored. Further investigation is required to establish the theoretical foundations necessary for integrated treatment approaches.

Lastly, many studies suffer from small sample sizes, which reduce statistical power and increase risk of bias. These limitations restrict the generalizability of findings and highlight the need for better-designed trials in future research.

### 4.3. Future Research Directions

Based on the current findings, upcoming research should prioritize the standardization of methodologies by developing consensus on protocol parameters, including session length and control conditions, to improve the reproducibility and comparability of NF studies. Large-scale RCTs are needed to evaluate the efficacy and safety of SCP, ILF, and ISF NF, particularly for ILF and ISF protocols, which remain poorly understood in pediatric populations. Future studies should expand the potential benefits of slow-oscillation NF interventions beyond disorders related to attentional control.

Research should also explore how NF can be integrated within multimodal treatment frameworks, including behavioral therapy and pharmacological approaches. Finally, future work should investigate factors that influence individual responsiveness to NF, including gender, cognitive abilities, motivation, and neurophysiological characteristics, to optimize treatment personalization and improve outcomes.

### 4.4. Conclusions

This narrative review explored the clinical efficacy of slow-oscillation NF protocols—SCP, ILF, ISF—in pediatric populations across conditions such as ADHD, ASD, tic disorders, epilepsy, and eating disorders. While SCP NF demonstrates preliminary efficacy in treating ADHD symptoms, findings across other conditions remain tentative due to methodological heterogeneity and limited data. Early results for ILF and ISF protocols are promising, especially in ASD, epilepsy and tic disorders, but require more rigorous evaluation.

Overall, the evidence base remains in its infancy. SCP NF currently has the strongest empirical support while ILF and ISF approaches are still emerging. Standardized protocols, larger trials, and an emphasis on individualized treatment responses will be essential to advancing the clinical utility of slow-oscillation NF in pediatric populations.

## Figures and Tables

**Table 1:** Overview of key features across slow-oscillation neurofeedback protocols.

	Slow Cortical Potentials (SCPs)	Infra-Low Frequency (ILF)	Infra-Slow Fluctuations (ISFs)
**Frequency Range**	Below 0.5 Hz	Below 0.1 Hz	Between 0.1–0.01 Hz
**Reflection of** **activity**	Neuronal	Neuronal and glial	Neuronal and glial
**Role**	Excitability shifts	State stabilization	Brain state transitions
**Applications**	Task-specific preparation	Arousal stabilization	State transitions
**Conscious** **reward**	Yes	Primarily non-conscious	Yes
**Electrode** **placements**	Unipolar (e.g., Cz with mastoid as reference)	Bipolar (e.g., T3–T4, with Cz as reference)	Montage varies by protocol
**Main equipment**	Sintered Ag/AgCl electrodes; Direct Current-coupled amplifier, PC Interface		

Note: This table summarizes the principal characteristics of three major slow-oscillation neurofeedback protocols (SCPs, ILF, ISFs), including their physiological targets, applications, and technical implementation. Ag/AgCl = silver/silver chloride; PC = personal computer. Electrode configurations refer to common but not exclusive setups and may vary by individual protocol and clinical goal. All protocols utilize the international 10–20 EEG system for electrode placement.

**Table 2:** Overview of studies on slow-oscillation neurofeedback (SO-NF) in pediatric populations.

Authors	Age	Diagnosis	*n*_(SO-NF)_ (Sex)	Design	Control	Protocol	Total NF Sessions	Clinical Change
Strehl et al. (2006)	8–13	ADHD	23 (4 F, 16 M)	Pre–Post-Assessment with 6-month follow-up	X	SCP	30	↑
Leins et al. (2006)	8–13	ADHD	19 out of 38 (3 F, 16 M)	RCT	✓	SCP	30	↑
Leins et al. (2007)	11–13	ADHD	19 out of 38 (3 F, 16 M)	Comparative study	✓	SCP	60	↑
Drechsler et al. (2007)	9–13	ADHD	17 out of 30 (4 F, 13 M)	RCT	✓	SCP	30	⟷ ↑
Doehnert et al. (2008)	9–12	ADHD	14 out of 26 (2 F, 12 M)	RCT	✓	SCP	30	↑
Gevensleben et al. (2009a)	8–12	ADHD	46 out of 72 (5 F, 41 M)	RCT	✓	SCP and θ/β	18 and 18	↑
Gevensleben et al. (2009b)	8–12	ADHD	59 out of 94 (8 F, 51 M)	RCT	✓	SCP and θ/β	18 and 18	↑
Gevensleben et al. (2010)	8–12	ADHD	59 out of 94 (8 F, 51 M)	RCT	✓	SCP and θ/β	18 and 18	↑
Wangler et al. (2011)	8–12	ADHD	59 out of 94 (8 F, 51 M)	RCT	✓	SCP and θ/β	18 and 18	↑
Christiansen et al. (2014)	7–11	ADHD	29 out of 58 (5 F, 24 M)	RCT	✓	SCP	30	⟷
Zuberer et al. (2018)	8–17	ADHD	48 (21 F, 27 M)	RCT	X	SCP	15 double sessions	↑ ⟷
Albrecht et al. (2017)	7–17	ADHD	24 (16 F, 8 M)	Pre–Post-Assessment	X	SCP	20	↑
Strehl et al. (2017)	7–9	ADHD	75 out of 144 (14 F, 61 M)	RCT	✓	SCP	25	⟷
Minder et al. (2018)	8–15	ADHD	38 out of 77 (15 F, 23 M)	RCT	✓	SCP	20–28	⟷
Baumeister et al. (2018)	10–13	ADHD	8 out of 16 (2 F, 6 M)	RCT	✓	SCP	20	↑
Baumeister et al. (2019)	11–13	ADHD	8 out of 15 (2 F, 6 M)	RCT	✓	SCP	20	↑
Aggensteiner et al. (2019)	7–9	ADHD	75 out of 150 (14 F, 61 M)	RCT	✓	SCP	25	↑
Okumura et al. (2019)	7–17	ADHD	22 (5 F, 17 M)	Pre-/Post Assessment	X	SCP	6	⟷
Heinrich et al. (2020)	8–12	ADHD	30 out of 48 (5 F, 25 M)	RCT	✓	SCP and θ/β	18 and 18	↑
Hasslinger et al. (2020)	9–13	ADHD	30 (9 F, 21 M)	qualitative study	X	SCP	25	↑
Aggensteiner et al. (2021)	7–9	ADHD	50 out of 103 (10 F, 40 M)	RCT	✓	SCP	25	⟷
H. Schneider et al. (2022)	7–21	ADHD	196 (41 F, 155 M)	Pre–/Post Assessment	X	ILF	~39	↑
Hasslinger et al. (2022a)	9–17	ADHD	51 out of 202 (13 F, 38 M)	RCT	✓	SCP	25	⟷
Hasslinger et al. (2022b)	9–17	ADHD	51 out of 202 (13 F, 38 M)	RCT	✓	SCP	25	⟷
Smith et al. (2016)	6, 29	ASD	2 (2 M)	case report	X	ISF	~ 80	↑
Rauter et al. (2022)	5	ASD	1 (1 M)	case report	X	ILF	26	↑
Konicar et al. (2021)	12–17	ASD	21 out of 41 (21 M)	RCT	✓	SCP	24	↑
Prillinger et al. (2022)	12–17	ASD	21 out of 41 (21 M)	RCT	✓	SCP	24	↑
Werneck-Rohrer et al. (2022)	12–17	ASD	12 (6 F, 6 M)	clinical trial	X	SCP	24	⟷
Klöbl et al. (2023)	12–17	ASD	21 out of 41 (21 M)	clinical trial	✓	SCP	24	⟷
Esmaeilzadeh Kanafgourabi et al. (2023)	12–16	ASD	12 out of 24 (12 M)	RCT	✓	ILF	15	↑
Legarda et al. (2011)	6–19	Epilepsy	3 (1 F, 2 M)	case report	X	ISF (ILF)	13–21	↑
Morales-Quezada et al. (2019)	10–19	Focal epilepsy	16 out of 44 (7 F, 9 M)	RCT	✓	SCP	25	↑
Prinz (2015)	14, 17	Tic Disorder	2 (1 F, 1 M)	case report	X	ILF	9–10	↑
Solberg and Solberg (2022)	8–18	Tic Disorder	100 (35 F, 65 M)	case studies	X	ILF	30–40	↑
Chirita-Emandi and Puiu (2014)	6–18	Obesity	12 out of 34 (5 F, 7 M)	pilot study	✓	ILF	20	↓ ↑

Note: Grey shading is used to indicate findings originating from the same underlying study. Only consecutive grey rows belong to the same base study. ADHD = Attention-deficit/hyperactivity disorder; ASD = Autism spectrum disorder; n_(SO-NF)_ = The number of participants receiving slow-oscillation NF (SO-NF) treatment relative to the total number of study participants; F = Female; M = Male; RCT = Randomized controlled trial; ✓ = Control condition included (e.g., sham, EMG feedback, group therapy); X = No control group; SCP = Slow cortical potentials; ILF = Infra-low frequency neurofeedback; ISF = Infra-slow fluctuation neurofeedback; θ/β = theta/beta; ↑ = Clinical improvement reported; ↔ = No significant clinical change reported; ↓ = Deterioration or less favorable outcome. Please note that a combination of ↑, ↔, and ↓ indicates that some symptoms have changed in one direction while others have changed towards another.

## Data Availability

No new data were created or analyzed in this study. Data sharing is not applicable to this article.
